# Optimized Ribonucleoprotein Complexes Enhance Prime Editing Efficiency in Zebrafish

**DOI:** 10.3390/ani15152295

**Published:** 2025-08-06

**Authors:** Lang Qin, Qiupeng Lin

**Affiliations:** 1 College of Fisheries, Southwest University, Chongqing 402460, China; qinlang040126@163.com; 2 State Key Laboratory for Conservation and Utilization of Subtropical Agro-Bioresources, Guangdong Basic Research Center of Excellence for Precise Breeding of Future Crops, College of Agriculture, South China Agricultural University, Guangzhou 510642, China; 3 Institute of BioFoundry, College of Life Sciences, South China Agricultural University, Guangzhou 510642, China

**Keywords:** aquaculture, genome editing, prime editor, zebrafish

## Abstract

Prime editing (PE) has emerged as a transformative genome editing technology, enabling precise base substitutions, insertions, and deletions without inducing double-strand DNA breaks (DSBs). However, its application in zebrafish remains limited by low efficiency. Here, we leveraged PE7, a state-of-the-art PE system, combined with La-accessible prime editing guide RNAs (pegRNAs), to enhance editing efficiency in zebrafish. By co-incubating PE7 protein with La-accessible pegRNAs to form ribonucleoprotein (RNP) complexes and microinjecting these complexes into zebrafish embryos, we achieved up to 15.99% editing efficiency at target loci—an improvement of 6.81- to 11.46-fold over PE2. Additionally, we observed 16.60% 6 bp insertions and 13.18% 10 bp deletions at the adgrf3b locus, representing a 3.13-fold increase over PE2. Finally, we used PE to introduce desired edits at the *tyr* locus, successfully generating zebrafish with the *tyr* P302L mutation that exhibited melanin reduction. These findings demonstrate that PE7 significantly enhances prime editing efficiency in fish, providing novel tools for functional gene studies and genetic breeding in aquatic species.

## Simple Summary

Scientists want to precisely edit genes in zebrafish to study how their bodies work and create better fish for farming. A tool called prime editing (PE) can make exact changes to DNA—like swapping, adding, or removing small pieces—without breaking both strands of DNA (which can cause uncontrollable gene mutations). But in zebrafish, PE usually does not work well, slowing down study progress. In this study, we tried to make PE more effective. We used a newer version, PE7 paired with La-accessible pegRNA, which is designed to work better with PE7. We injected this pegRNA into zebrafish embryos. The results show that we boosted the editing success to up to 15.99% at target sites—6 to 11 times better than older PE methods. We also created zebrafish with a specific mutation (tyr P302L) that reduces melanin (the pigment that gives color), a trait scientists struggled to make before. These findings mean that PE7 makes gene editing in zebrafish much more reliable. By improving this tool, researchers can better understand fish biology and develop new, improved fish for aquaculture—helping both science and the farming of aquatic animals.

## 1. Introduction

Genome editing technologies are indispensable tools for elucidating gene functions and generating desirable traits in aquatic organisms [1,2,3,4,5,6]. Among these, the CRISPR/Cas9 system has emerged as a revolutionary platform for genetic improvement in aquatic animals, owing to its high efficiency and ease of use [7,8,9]. However, genome editing mediated by non-homologous end joining (NHEJ) via CRISPR/Cas9 is inherently stochastic, often producing unpredictable insertions or deletions (indels) that hinder the precise modulation of target traits [10]. While homology-directed repair (HDR) based on nucleases enables precise editing, its reliance on exogenous donor DNA templates and generally low efficiency severely limit large-scale applications [11]. In recent years, base editors (BEs) have provided novel tools for single-nucleotide substitution. Composed of nCas9 (nicked Cas9) fused with cytosine deaminases (e.g., APOBEC) or adenine deaminases (e.g., TadA), BEs enable direct C•G→T•A (cytosine base editors, CBEs) or A•T→G•C (adenine base editors, ABEs) substitutions without requiring double-strand breaks (DSBs) or donor templates [12,13,14]. These tools have been successfully applied to precisely modify traits such as pigmentation phenotypes and metabolic pathways in model organisms like zebrafish [15,16,17,18,19]. Nevertheless, BEs remain constrained to single-nucleotide substitutions, lacking the capacity for base transversion (e.g., C→G/A→T) or small-indel generation, thus failing to meet the demands of complex trait engineering.

In 2019, the development of PE introduced a transformative solution to these limitations. Unlike conventional tools, PE operates without DSBs or donor DNA templates and is theoretically capable of generating all 12 types of single-nucleotide substitutions as well as arbitrary small indels, significantly expanding both the scope and accuracy of genome editing [20]. The PE system comprises a nCas9 (H840A), an engineered reverse transcriptase (MMLV-RT), and pegRNA [12]. Its operational mechanism proceeds as follows: First, nCas9 introduces a single-strand break (SSB) in the non-target DNA strand at the target locus, generating a single-stranded DNA (ssDNA) intermediate. Next, the pegRNA—containing a Primer Binding Site (PBS) and a Reverse Transcription Template (RTT)—hybridizes to this ssDNA to prime reverse transcription. The RTT is reverse-transcribed and transferred to the target strand, forming a DNA/RNA hybrid intermediate. Finally, RNase H degrades the RNA component, exposing a DNA flap that is integrated into the genome via endogenous DNA repair mechanisms (e.g., homologous recombination or NHEJ), thereby achieving precise editing [12]. Although PE has been demonstrated to function efficiently in animals (e.g., mice, human cells), plants (e.g., rice, Arabidopsis), and model fish (e.g., zebrafish) [21,22,23,24,25,26], its editing efficiency in fish remains extremely low, with applications currently limited to zebrafish or other non-model fish, severely restricting its utility in aquatic genetic breeding.

Researchers have developed multiple strategies to enhance PE efficiency. For instance, Doman et al. applied phage-based continuous evolution (PACE) to engineer PE6 [27]. Nelson et al. added a motif at the 3′ end of pegRNA to prevent the degradation of pegRNA to improve editing efficiency [28]. Yan et al. identified La, a small-molecule-binding protein critical for the process of prime editing, fused it with PEmax to generate PE7, and found that adding polyU to pegRNA’s 3′ end enhances PE7 interaction and editing efficiency—this pegRNA was termed La-accessible pegRNA by them [29]. Beyond engineering PE and pegRNAs, Lin et al. introduced a dual-pegRNA strategy, using two distinct pegRNAs to target the same locus and boost editing efficiency [30].

**Figure 1:**
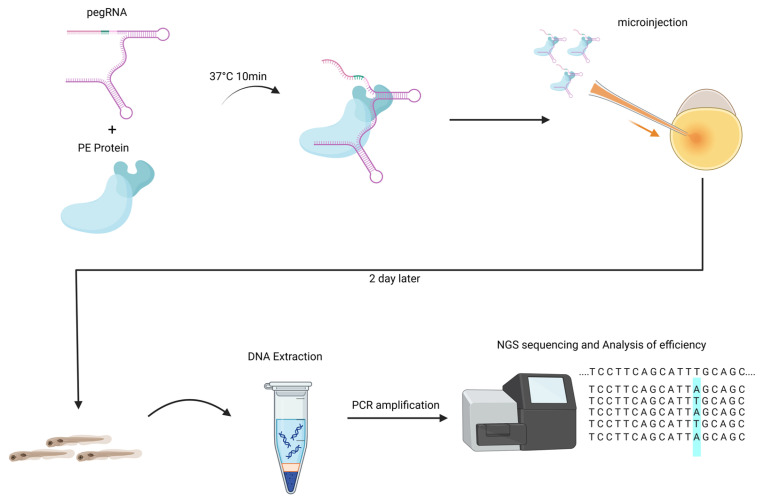
Workflow for RNP injections into zebrafish embryos for PE and analysis of editing efficiency. The blue highlight represents the editing area.

Here, we utilized PE7 and La-accessible pegRNA to perform genome editing at multiple loci, achieving single-base substitutions or small indels in zebrafish. We incubated prime editing (PE) with pegRNA to form the PE RNP complex, then microinjected this complex into zebrafish embryos. At 2 days post-fertilization (dpf), genomic DNA was extracted from the embryos, amplified using barcoded primers, and subjected to next-generation sequencing (NGS) for editing efficiency analysis (Figure 1). The maximum editing efficiency reached 16.60%, which was 6.81- to 11.46-fold higher than that of PE2. Notably, we generated visibly reduced-pigmentation zebrafish harboring the *tyr* P302L mutation (CCC→CTC) for the first time. This study not only establishes a robust PE framework for aquatic genetic engineering but also paves the way for precise trait modulation in commercially important species.

## 2. Materials and Methods

### 2.1. Ethical Statement

All experimental procedures were conducted in compliance with the guidelines of the Animal Welfare and Ethics Committee of Hunter biotechnology, Inc. (Hangzhou, China) and were approved by the committee (IACUC-2025-12465-01).

### 2.2. Zebrafish Maintenance

Wild-type AB strain zebrafish eggs were incubated at 28.5 °C in a humidified incubator. Adult fish were maintained in a recirculating aquaculture system at a density of 30 individuals per 3 L tank, with an age range of 6–15 months. Water parameters (pH 7.0–7.5, ammonia <0.1 ppm, nitrite <0.05 ppm) were monitored daily, and the tanks were cleaned weekly to ensure optimal rearing conditions. All zebrafish in this study are breeding at Hunter Biotechnology, Inc. (Hangzhou, China).

### 2.3. pegRNA Generation

All pegRNAs were chemically synthesized by GenScript Biotech (Rijswijk, The Netherlands) with 5′ and 3′ modifications (methylated or phosphorothioate linkages) to enhance stability. Sequences of all pegRNAs and the detailed modification in every pegRNA are provided in Table S1. Lyophilized pegRNAs were resuspended in nuclease-free water to a final stock concentration of 1000 ng/μL and stored at −80 °C until use.

### 2.4. PE RNP Preparation and DNA Extraction

**Figure 2:**
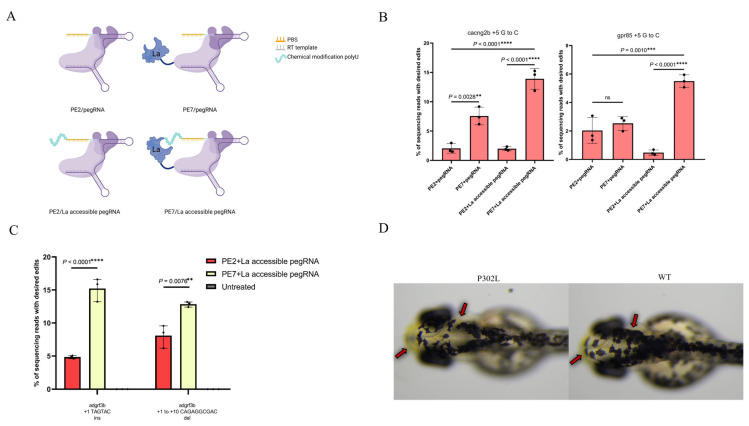
Optimized prime editors for higher-efficiency genome editing in zebrafish. (**A**) A diagram of the PE protein and pegRNA complex in this study. (**B**) The editing efficiency of optimized prime editors on single-point mutations. (**C**) The editing efficiency of optimized prime editors on small indels. (**D**) Head images of a zebrafish with the *tyr* P302L mutation and a wild-type zebrafish. The frequencies (mean  ±  s.e.m.) in B and C were calculated from three independent experiments (*n*  =  3). *p* values were obtained using one-way analysis of variance. ^ns^ *p* > 0.05, * *p*  <  0.05, ** *p*  <  0.01, *** *p*  <  0.001 and **** *p*  <  0.0001. The red arrow in the figure indicates the site of melanin disappearance.

At the one-cell stage of zebrafish embryos, 2 nl RNP complexes were microinjected into the yolk cytoplasm. Each RNP complex contained 750 ng/μL PE2 or PE7 nuclease and 240 ng/μL pegRNA or La-accessible pegRNA (Figure 2A). For developmental stage synchronization, injected embryos were raised at 28.5 °C. At 2 days post-fertilization (dpf), 6–8 normally developed embryos from each experimental group were collected, and genomic DNA was extracted using the QIAamp DNA Mini Kit (Qiagen, Hilden, Germany) following the manufacturer’s protocol. The genomic DNA was stored at −20 °C for downstream sequencing.

### 2.5. Microinjection and Image Acquisition in Zebrafish

For the *tyr* P302L injection group, the embryos were anesthetized using 0.03% Tricaine (Sigma-Aldrich, Saint Louis, MO, USA) and carefully mounted in 4% methylcellulose at 2 dpf. Imaging was conducted using either an XM10 digital camera (OLYMPUS, Tokyo, Japan) or AxioCam MRc5 digital camera (Leica, Wetzlar, Germany) on an SZX2-FOF microscope (OLYMPUS). Post-capture adjustments and enhancements were made using Adobe Illustrator software (v10.1.1).

### 2.6. Deep Amplicon Sequencing and Data Analysis

Amplicon sequencing was repeated three times for each target site using genomic DNA extracted from three independent samples. In the first round of PCR, the target region was amplified from genomic DNA with site-specific primers. In the second round, both forward and reverse barcodes were added to the ends of the PCR products for library construction (all primers used here can be found in Table S2). Equal amounts of PCR products were pooled and sequenced commercially (Novogene) using the illumina Novaseq X plus platform, and the pegRNA target sites in the sequenced reads were examined for substitutions and indels. All data were processed with GraphPad Prism 8.0.1 (GraphPad Software, Inc., San Diego, CA, USA). Data from at least three distinct experiments are provided as mean  ±  SD. Independent sample *t*-tests were employed to determine significant differences in mean values between the two groups. The mean values of multiple groups were compared using one-way ANOVA (and nonparametric or mixed) tests. For all tests, the statistical significance criteria were set at *p*  < 0.05.

## 3. Results

### 3.1. Optimized PE Significantly Improves the Efficiency of Prime Editing

To enhance the efficiency of PE in zebrafish genome editing, we incubated the most efficient prime editor, PE7, with its corresponding pegRNAs, followed by microinjection into zebrafish embryos. PE2/pegRNA RNPs were also injected as controls. The results showed that at the *cacng2b* locus, PE7 achieved an editing efficiency of 7.57%, which was 2.75-fold higher than that of PE2. However, at the *gpr85* locus, no significant difference in editing efficiency was observed between PE7 and PE2.

Previous studies have demonstrated that the small protein La in PE7 interacts with the polyU motif at the 3′ end of RNA, and chemical modification of this polyU tail can enhance such interactions. Therefore, we synthesized La-accessible pegRNAs with a chemical modification to the polyU tail (UUmUmU*mUU) that interacts most strongly with the La protein. The results indicated that La-accessible pegRNAs significantly enhanced editing efficiency, with PE7 showing a 6.98- to 11.46-fold increase compared to PE2. Additionally, the efficiency of the PE2/La-accessible pegRNA combination was reduced (Figure 2B).

### 3.2. Short Indels in Zebrafish

Indels in the genome induce frameshift mutations, altering gene function. Unlike CRISPR/Cas9, which rely on NHEJ to induce a random indel, PE theoretically enables the precise introduction of indels of any length, offering a more versatile tool for gene function studies. To evaluate the efficiency of PE7 in generating short indels in zebrafish, we designed pegRNAs to induce 6 bp insertions and 10 bp deletions at the adgrf3b locus. This approach achieved 13.18% 6 bp insertions and 16.6% 10 bp deletions, representing 1.66- to 1.69-fold higher efficiency compared to PE2-mediated indel generation (Figure 2C).

### 3.3. Editing Efficiency at Complex Disease Mutation tyr P302L

Finally, we evaluated the editing efficiency of PE7 at the complex disease mutation site *tyr* P302L, which cannot be precisely generated by BEs or CRISPR/Cas9 due to the presence of multiple CpG sites. Although PE2 can achieve this substitution, prior studies in zebrafish only identified germline-transmitted mutant alleles without observable phenotypes due to low editing efficiency. By utilizing PE7 combined with La-accessible pegRNAs, we successfully induced this mutation, leading to visibly reduced pigmentation in the zebrafish head (Figure 2D).

## 4. Discussion

PE has revolutionized genome engineering by enabling precise single-base substitutions, insertions, and deletions without inducing DSBs. While previous work has successfully realized prime editing with PE2 RNP in zebrafish [26], further improvements in editing efficiency remain necessary. Here, we utilized PE7 and La-accessible pegRNAs to achieve efficient genome editing in zebrafish embryos, establishing a zebrafish albinism model and providing novel tools and examples for aquatic animal breeding.

First, we demonstrated that the delivery of PE7 RNP is a novel approach to enhance the editing efficiency in zebrafish. By microinjecting PE7 and a pegRNA RNP complex into zebrafish single-cell stage embryos, we observed that PE7 achieved an editing efficiency of 7.57% at the *cacng2b* locus—2.75-fold higher than PE2. However, no significant difference in editing efficiency was detected between PE7 and PE2 at the *gpr85* locus.

Given that PE7 comprises an engineered PE2 fused with a small-molecule La protein, the lack of efficiency enhancement at the *gpr85* locus may be attributed to two potential factors: the inherent insensitivity of the engineered PE2 to this specific locus or failure of the La protein to augment editing efficiency. Previous studies have demonstrated that the La protein in PE7 interacts with the polyU motif at the 3′ end of pegRNAs, and the chemical modification of this polyU tail can strengthen these interactions [31]. We hypothesize that enhancing PE7-pegRNA interactions via polyU-modified pegRNAs will significantly improve editing efficiency.

To validate this hypothesis, we synthesized La-accessible pegRNAs bearing a chemically modified polyU tail (UUmUmU*mUU) [29]. The results demonstrated that these La-accessible pegRNAs significantly enhanced editing efficiency: PE7 mediated a 6.98- to 11.46-fold higher efficiency compared to PE2 (Figure 2B). This not only confirmed the superior performance of PE7 but also indicated that pegRNAs used with PE7 should ideally contain a polyU tail. Additionally, we evaluated PE7’s capability of generating small indels, achieving a 3.1-fold improvement in small-indel induction compared to PE2 (Figure 2C).

Notably, the PE2/La-accessible pegRNA combination exhibited a moderate reduction in efficiency at the *gpr85* locus (Figure 2B). Previous studies have demonstrated that appending complex tertiary structures—such as tevopreQ1 or viral exoribonuclease-resistant RNA motifs (xrRNAs)—to the 3′ end of pegRNAs can protect the 3′ end from degradation, preventing truncated pegRNAs from outcompeting intact pegRNAs and thereby enhancing PE2 efficiency [32,33]. Theoretically, adding a polyU structure to the 3′ end of pegRNAs might also improve editing efficiency. However, our results showed a decrease in editing efficiency at the *gpr85* locus, whereas at the *cacng2b* locus, efficiency remained comparable to that of pegRNAs without polyU. We hypothesize that this discrepancy may arise from two factors: the protective effect of the polyU tail was insufficient to prevent truncation, or it conversely impaired the binding and editing efficiency of PE2-pegRNA complexes at the target site. However, the specific mechanism still awaits further clarification.

**Figure 3:**
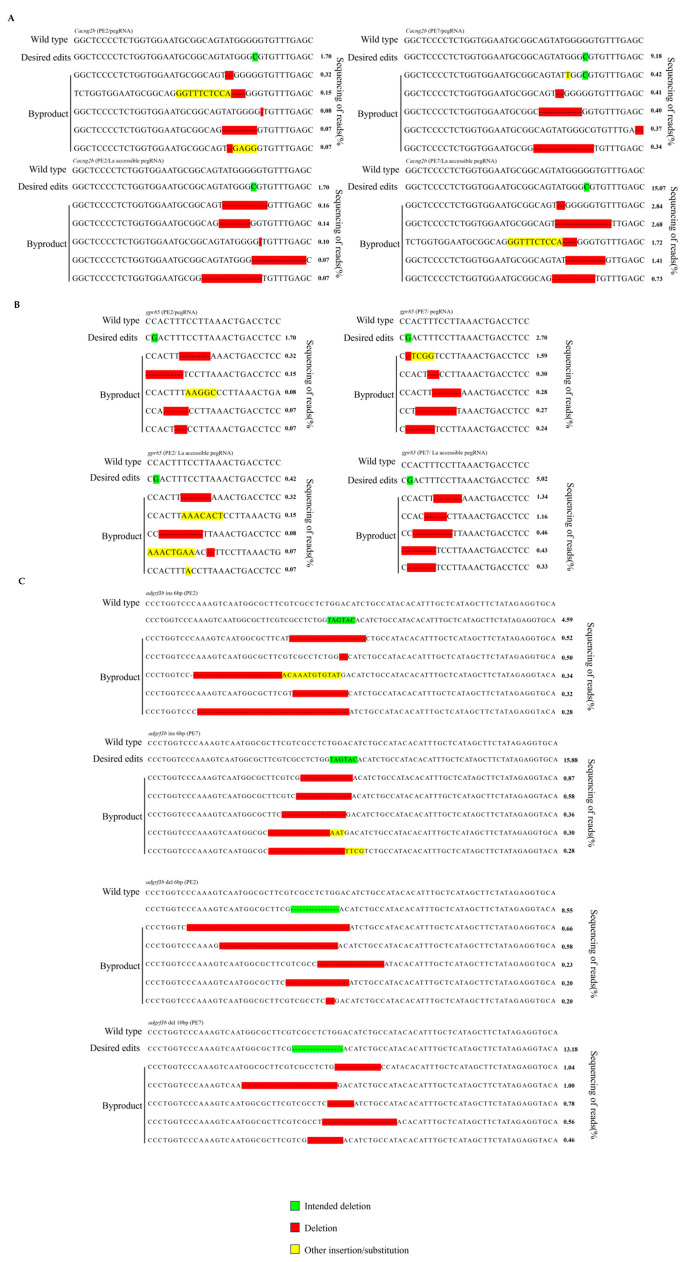
Frequencies of desired edits and byproducts sequencing reads. (**A**) Frequencies of desired edits and byproducts sequencing reads in cacng2b sites. (**B**) Frequencies of desired edits and byproducts sequencing reads in gpr85 sites. (**C**) Frequencies of desired edits and byproducts sequencing reads in adgrf3b sites.

Finally, we applied PE7 to generate zebrafish with the pathogenic mutation *tyr* P302L (CCC→CTC). This mutation is adjacent to multiple cytosine sites, rendering it refractory to precise induction by traditional CRISPR/Cas9 systems and Bes [34]. Although PE2 can achieve this substitution, prior studies in zebrafish only detected germline-transmitted mutant alleles without observable phenotypes [26]. In this study, by injecting PE7 RNP complexes into zebrafish embryos, we generated zebrafish harboring the *tyr* P302L mutation with significantly reduced skin melanin for the first time (Figure 2D). This finding not only validated PE7’s capability to induce single-base substitutions at complex loci but also established the first zebrafish skin albinism model using prime editing technology, providing an ideal tool for elucidating pathogenic mechanisms and drug screening. Additionally, we analyzed desired edits and byproducts across different edit sites (Figure 3). Our results demonstrated that while PE7 exhibited significantly higher efficiency than PE2, byproducts remained rare, further confirming the superiority of PE7/La-accessible pegRNAs.

Although RNP-based PE7 significantly improved PE efficiency in zebrafish, there remains substantial room for further enhancement. In this study, all PBS sequences were designed to be 13 nt, a length widely considered optimal for PE [12]. However, a recent study revealed that the best PBS lengths differ significantly between PE systems delivered via RNP/mRNA versus plasmid transfection: 6 to 7 nt PBS sequences have been shown to strikingly enhance editing efficiency in RNP-PE and mRNA-PE systems [35]. Additionally, researchers have observed that refolding pegRNAs can increase editing efficiency at the *cacng2b* locus to 8%, suggesting that such structural modifications to pegRNAs may also provide a strategy to improve PE7 efficiency [36].

In this study, PE RNP was used to deliver the editor into zebrafish, but plasmid delivery and mRNA delivery are also highly efficient methods. So far, there have been no studies on PE in zebrafish or non-model fish using plasmids or mRNAs, possibly because these methods are less efficient [4]. Perhaps the efficiency of PE in fish can be further enhanced through methods such as codon optimization and the modification of PE elements.

## 5. Conclusions

In conclusion, our research indicates that the gene editing ability of PE7 in fish is significantly enhanced compared to PE2, providing a powerful tool for the study of fish gene functions and genetic breeding.

## Data Availability

The data presented in this study are available in NCBI (PRJNA1288536).

## Supplementary Materials

The following supporting information can be downloaded at: https://www.mdpi.com/article/10.3390/ani15152295/s1, Table S1: Target sequences, 3′ extension sequences, and chemically modified parts of pegRNA; Table S2: PCR primers used for deep sequencing in this study.
